# The Yemeni genetic structure revealed by the Y chromosome STRs

**DOI:** 10.1007/s12024-025-00975-z

**Published:** 2025-03-01

**Authors:** Khalid Al-Shoba, Nabil Al-Hamadi, Eida Khalaf Almohammed, Sibte Hadi, William Goodwin, Hayder Lazim

**Affiliations:** 1 Department of Forensic Medicine and Clinical Toxicology, Faculty of Medicine and Health Sciences, Sana’a University, Sana’a, Yemen; 2 Ministry of Interior of Qatar, Doha, Department of Biomedical Sciences, College of Health Sciences, QU Health, , Qatar University, Doha, Qatar; 3 Naif Arab University for Security Sciences, Riyadh, Saudi Arabia; 4 School of Law and Policing, University of Central Lancashire, 135A Adelphi St, Preston, PR1 7BH UK; 5 Faculty of Health, Social Care and Medicine (FHSCM), School of Medicine, Edge Hill University, Ormskirk, L39 4QP UK

**Keywords:** Yemen, Y-STR, Y-haplogroup, Phylogenetics, Allelic richness, Ancestry variability

## Abstract

Yemen, with its rich historical background and strategic geographical position at a major crossroads of trade and migration, offers an ideal setting for exploring population genetics. This study aimed to develop a Y-STR database for a Yemeni population and compare it with existing regional databases in the Middle East. For this investigation, buccal swabs were collected from 128 unrelated males. Genomic DNA was extracted using the QIAamp^®^ DNA Mini Kit, and Y-chromosomal STR profiling was performed with the AmpFℓSTR^®^ Yfiler™ PCR Amplification Kit to generate haplotype data across 17 Y-STR loci. The final dataset exhibited a haplotype diversity of 0.008 and a discrimination capacity of 0.95. Among the STR loci assessed, DYS458 emerged as the most polymorphic, displaying a gene diversity of 0.87 and accounting for the majority of microvariant alleles (62.5%). Additionally, haplogroup analysis using the NevGen haplogroup predictor tool revealed two predominant haplogroups within this Yemeni population: J1a (59.37%) and E1b1b (21.09%). Comparisons with 52 Middle Eastern populations (encompassing 5,568 individuals) through multidimensional scaling, phylogenetic assessments, admixture analyses, and ancestry variability evaluations collectively underscore the unique genetic landscape of Yemen. Overall, the combined findings indicate evidence of a potential founder effect within the Yemeni population. Taken together, these data not only enrich the forensic and population genetic understanding of the region but also emphasize Yemen’s pivotal role in illuminating migration and demographic processes in the Middle East.

**Supplementary Information:**

The online version contains supplementary material available at 10.1007/s12024-025-00975-z.

## Introduction

Y chromosome short tandem repeats (Y-STRs) are pivotal in forensic science and population genetics due to their ability to trace paternal lineages and provide male-specific genetic information. In forensic applications, Y-STRs are particularly useful in resolving cases involving male perpetrators, such as sexual assaults, where mixed male and female DNA samples are present [1]. The Y chromosome’s non-recombining nature allows Y-STRs to track direct paternal ancestry, offering insights into genealogical connections and paternal lineage [2]. Additionally, Y-STR databases enhance the effectiveness of forensic analysis by compiling haplotype frequencies from diverse populations, which aids in the statistical interpretation of DNA evidence [3]. These databases are crucial for ensuring accurate matches and supporting legal proceedings by providing a reference framework for evaluating the rarity or commonality of a given Y-STR profile within specific populations. Moreover, Y-STR data contribute significantly to population genetics by elucidating patterns of human migration and genetic diversity across different demographic groups [4].

**Fig. 1:**
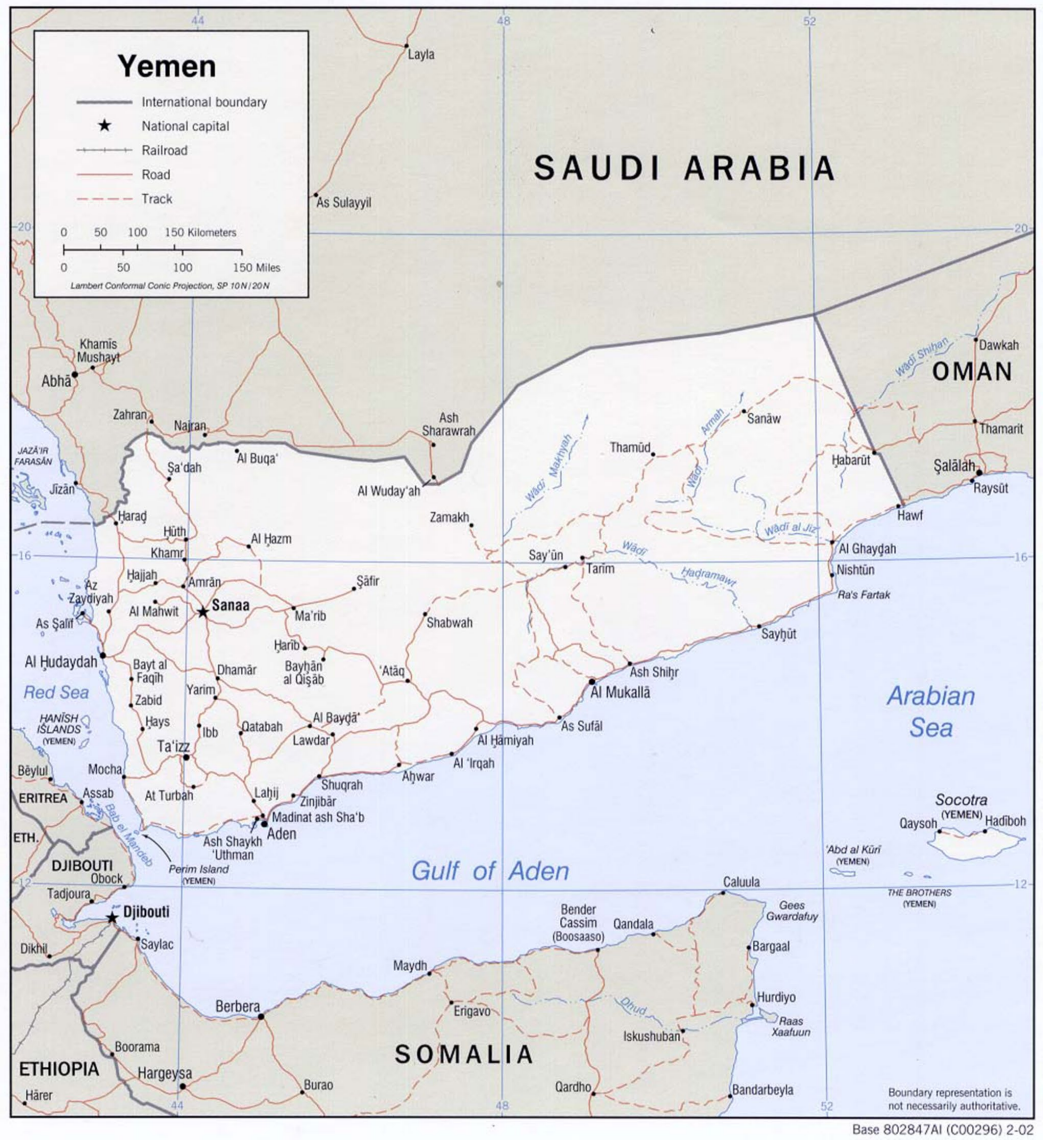

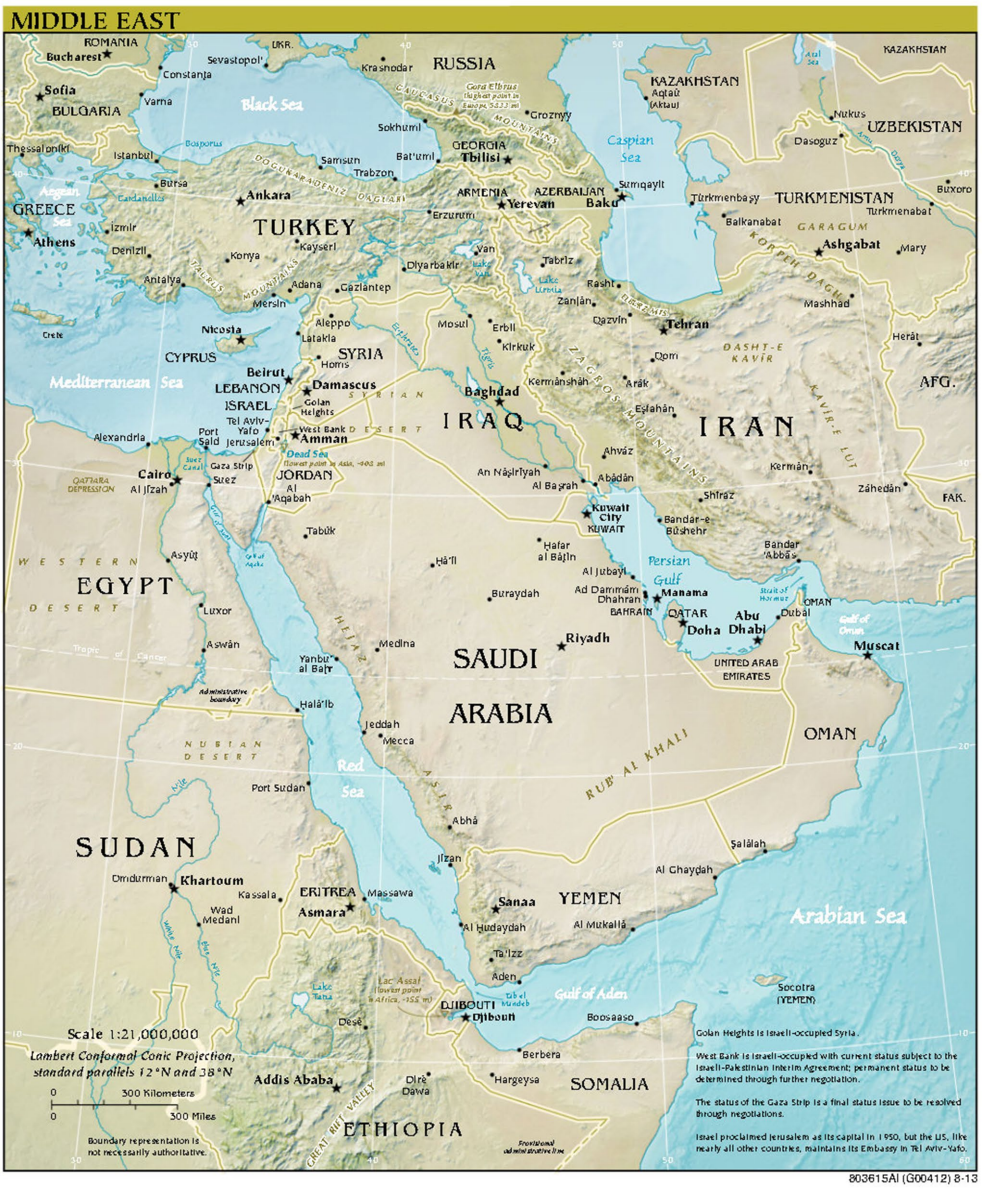
Map representing the geographic location of (**a**) The middle East (**b**) Yemen. Courtesy of the University of Texas Libraries, The University of Texas at Austin [9]

With an estimated population of 35.2 million in 2024 [5], Yemen (officially the Republic of Yemen, and historically Arabia Felix) is located at the south-western part of the Arabian Peninsula [6]. While most Yemenis are Arabs, there are immigrants from Africa and south Asia. Yemen has a long maritime border that extends from the southern part of the red sea on the west to Gulf of Aden and Arab Gulf on the south. The strategic location of Yemen overseeing Bab El-Mandeb strait, which separates the Arabian Peninsula from the horn of Africa and the red sea from the Indian ocean, has made it attractive as a route for trade and migration and for colonization by different powers/empires (Ethiopian, Persian, Ottoman, British, etc.). Figure 1 presents maps detailing the geographical layout of the Middle East and Yemen.

The history of Yemen is estimated to begin around 2000 years BC [7], and has harboured some of the oldest civilisations in the region, namely; Maean, Sabaean and Himyarite, partly due its mountainous fertile soil and rich water sources leading to abundance of grains, fruits and coffee. (hence the name Arabia Felix) and partly because of its location at trade cross route [7, 8]. In addition to their migration for trade or war, it is widely believed, by Yemenis, that after the collapse of the Great Dam of Marib, which was built around 1750 BC and sustained life of the Yemenis and their agriculture, Yemeni people migrated out of Yemeni to the neighbouring regions [8].

Overall, the integration of Y-STRs and comprehensive databases underpins both the forensic investigation process and the broader understanding of human genetic history.

In this study, we present a comprehensive database comprising 17 Y-STR loci specific to the Yemeni population, which we are introducing to the scientific community. Furthermore, we examined the genetic relationships between Yemen and broader Middle Eastern populations.

## Materials and methods

### Collection of Yemeni population samples

Buccal swabs were collected from 128 unrelated male Yemeni students at the Faculty of Medicine and Health Sciences, Sana’a University, Yemen. All samples used in this study were obtained for the purpose of the research and ethical approval was granted by the ethical committee of the Faculty of Medicine and Health Sciences, Sana’a University, on 3rd of April 2007. Informed consent forms were completed by all participants and authors had no access to information that could identify individual participants during or after data collection. The data were collected in April 2007 and made accessible for research purposes in June 2007.

### DNA extraction

DNA was extracted from the buccal swabs using a modified QIAamp^®^ DNA Mini Kit protocol (Qiagen™ Ltd., Crawly, West Sussex). To monitor contamination, a negative extraction control was processed with each batch of samples.

Extracted DNA was subjected to electrophoresis in 0.8% agarose gel. Then, it was stained in an ethidium bromide solution (25 ng/500 mL H_2_O) for 10–20 min. After that, DNA was visualised on a UV transilluminator. Quantification was done via comparison of the intensity of unknown DNA bands to that of a set of known standard lambda DNA run concurrently with the samples.

### Yfiler^TM^ PCR amplification and fragment analysis

The Yfiler™ kit was used to generate 17 loci: DYS19, DYS389I, DYS389II, DYS390, DYS391, DYS392, DYS393, DYS385a, DYS385b, DYS438, DYS439, DYS437, DYS448, DYS456, DYS458, DYS635, and YGATA-H4.

DNA fragment amplification was carried out in thermal cycler microtubes (Thermo Fisher Scientific) on a PCR 2700 system (Applied Biosystems, Foster City, USA) from about 0.4 ng of template DNA using the AmpFℓSTR^®^ Yfiler™ PCR amplification kit (Applied Biosystems, Warrington, Cheshire) as per the kit manufacturer’s recommendations except for using a third of the recommended reaction volume. Each batch of samples run on a PCR included a PCR positive control consisting of AmpFℓSTR^®^ DNA 007 at a concentration of 0.1 ng/µl and a PCR negative control consisting of a female AmpFℓSTR^®^ DNA 9947 A (10 ng/µl).

Amplified DNA products of AmpFℓSTR^®^ Yfiler™ loci were capillary electrophoresed using the ABI PRISM^®^ 310 Genetic Analyser (Applied Biosystems) using capillary filled with CE310 optimised polymer 4 (POP4) (Applied Biosystems, Warrington, Cheshire). Fragment analysis was conducted utilizing GeneMapper™ ID Software version 3.2 (Applied Biosystems), which also facilitated the designation of alleles at the loci by comparing them with the Yfiler™ allelic ladder. Additionally, reagent blanks were processed concurrently with each batch of samples to ensure the accuracy and reliability of the analyses.

## Statistical analyses

### Forensic and population genetic parameters

The haplotype diversity for the Yemeni population samples was evaluated by Nei’s formula [10], HD = n*(1 − Σ pi^2^)/ (*n* − 1) where n is the sample size and pi is the ith’s haplotype frequency. Haplotype frequency was calculated by the counting method. Genetic diversity (GD) was calculated as 1 − Σ pi^2^, where pi is the allele frequency. The match probability (MP) was calculated as Σ pi^2^, where pi is the frequency of the ith haplotype. The STRAF online tool was used to calculate haplotype diversity, GD, and MP [11]. Discrimination capacity (DC) was calculated by dividing the number of different haplotypes (h) by the total number of samples in a certain population (n) using the following formula: DC = h/n [12]. The haplotype match probability (HMP) was calculated as HMP = 1– HD [13]. The Yemeni population data were obtained using Y-STRs based on the Yfiler^™^ kit, and compared to available published data for other close and distant populations.

The population genetic structure in our data was evaluated by the analysis of molecular variance (AMOVA). Molecular data were obtained for the Yemeni population using Y-STRs based on the Yfiler^™^ System and compared with the available data on other Middle Eastern populations [3, 14–27]. Comparison with other datasets required reduction of the number of STRs to a shared set of 17, so that more Middle Eastern populations could be included in this analysis. Arlequin 3.5.2.2 software [28, 29] was used to calculate the average pairwise differences between (PiXY) and within populations (PiX), in addition to the corrected average pairwise difference between populations (PiXY − (PiX + PiY)/2). More specifically, genetic distances between groups of males were quantified by R_ST_ calculations based on Y-STR data and multi-dimensional scaling (MDS) plots. MDS analysis is also used to investigate genetic similarities between populations [30], and to visualize the variances of the genetic differences in Y-STR and between populations. The genetic matrices plots, the MDS plot and the phylogenetic tree were generated by using R statistical software version 4.0.

### Yemeni Y haplogroup assignment

In this research study, the methodology employed involved the use of full Y17 haplotypes to allocate haplotypes to their most likely haplogroup, a process which was facilitated through the online Y-DNA Haplogroup Predictor NevGen [31]. This online tool based on the Bayesian approach which is previously-implemented by the Whit Aethy’s Haplogroup Predictor program [32].

### Median-joining networks Y-STR haplogroups analysis

To gain a deeper comprehension of the association between Y-STR haplotypes and their corresponding predicted haplogroups, a median-joining network was constructed, integrating the haplotypes from our dataset. This exercise served to illustrate the consistency between haplogroup prediction and haplotype clustering. The implementation of this approach involved the utilization of two software applications, specifically Network v5.0 and Network Publisher v2.1.2.5 [33].

To ensure the precision and dependability of the outcomes, intermediate alleles containing repeat numbers were rounded to the nearest integer. Adhering to the author’s guidance, the constitutively duplicated loci (specifically, DYS385 a and b) were excluded from the network structure. It is important to highlight that any absent data or removed alleles were substituted with the code ‘99’ in the input files, in accordance with the customary procedure for treating such data as missing.

### Allelic richness in different middle Eastern populations

To achieve a more comprehensive understanding of genetic diversity and population relationships, the study investigated allele distributions across Middle Eastern populations. Specifically, the study focused on evaluating the number of distinct alleles within each population, as well as the presence of alleles exclusive to a particular population, not observed in any other populations. These fundamental characteristics are valuable indicators when examining populations at a specific locus, especially in the analysis of highly variable multiallelic markers like microsatellites. The Allelic Diversity Analyzer (ADZE) Version 1.0 software was utilized to accurately quantify the count of distinct and private alleles [34, 35].

### Admixture analysis

This study looked at the population admixture of 52 (5568 individual) Middle Eastern populations [3, 14–27] using the programme STRUCTURE version 2.3.7 and an admixture model [36, 37]. The computational parameters were configured to include 100,000 burn-in iterations followed by 10,000 Markov Chain Monte Carlo (MCMC) repetitions, conducted over 10 independent runs for each value of K.

The output was processed using the STRUCTURE HARVESTER program to evaluate the probability values across a wide range of K values and determine the optimal number of genetic clusters that best fit the data [38, 39]. To consolidate the findings, multiple iterative analyses of each dataset were aligned using CLUMPP [40, 41], These aligned results were then utilized to create the population Q-matrix graph using Distruct [40, 42].

Ancestry variability among Middle Eastern populations was evaluated by examining the degree of variation in membership coefficients generated by the STRUCTURE program. The analysis of membership coefficients, differentiating between admixed and non-admixed individuals across six designated clusters, was conducted utilizing the FSTruct program [43, 44].

## Results

The 17 Y-STR loci profiled with AmpFℓSTR^®^ Yfiler™ kit were amplified for 128 Yemeni males. S1 Table contains a full list of Yemeni haplotypes, as well as other sample information; data are also accessible from YHRD, release R69 (Accession number YA005529).

### Y-STR alleles and haplotype diversity within the Yemeni population

**Fig. 2:**
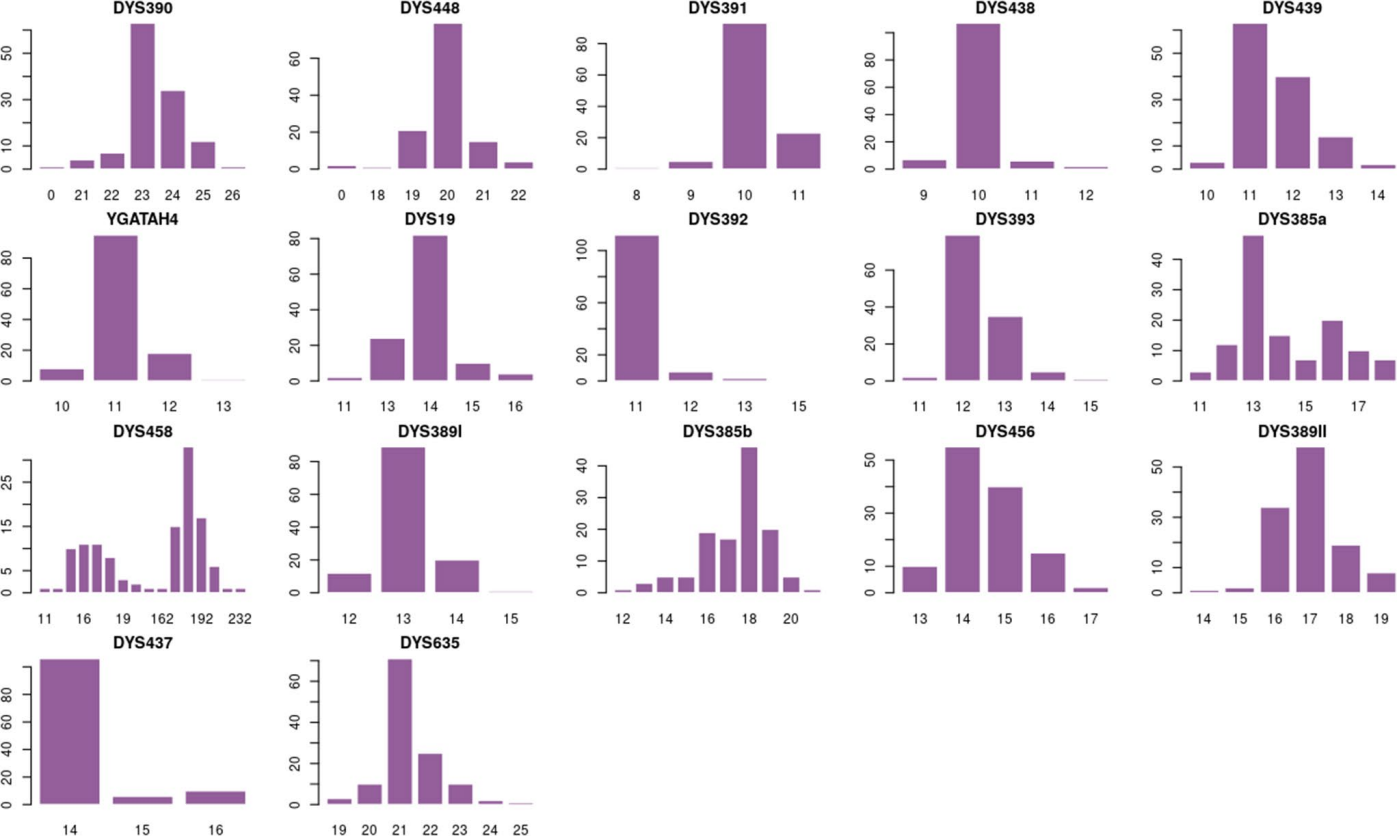
The alleles frequencies of the AmpFℓSTR^®^ Yfiler™ loci in the Yemeni population. The difference between the overall repeat number at DYS389II and the repeat number at DYS389I was used to encode DYS389II alleles

Our analysis revealed 128 haplotypes and 122 (91.4%) unique haplotypes. The Fraction of Unique Haplotypes (FUH) was 95.9 with only 5 haplotypes shared by 11 individuals. The frequency of the most common haplotype was 0.02459, shared by 3 individuals. The kit has a discrimination capacity of 0.953125. Allele frequency distributions of the 17-STR loci and the most frequent allele for each locus are presented in S2 Table and Fig. 2 for the 128 males of the population under study.

While the haplotype diversity (HD) of our population, as measured using the AmpFℓSTR^®^ Yfiler™ kit, was 0.008, the average gene diversity was 0.530 (SD = 0.208). The locus DYS458, with 16 alleles, has the highest gene diversity and power of discrimination, and the lowest match probability 0.868446, 0.861327, 0.138672, respectively. The locus DYS392, with a total of four alleles, had the lowest gene diversity and power of discrimination, and the highest match probability 0.154857, 0.153587, 0.846412, respectively. The number of loci with 5 or more alleles was 11 (S3 Table). The entirety of microvariant alleles was identified at the locus DYS458, constituting 80 haplotypes with a prevalence of 62.5%.

### Haplogroup prediction results for Yemeni samples

Two main haplogroups present in our dataset the J1a haplogroup (constituting 59.37% of the total sample), and the E1b1b haplogroup (constituting 21.09% of the total samples). It was observed that 76 of the microvariant alleles were linked to the J1a haplogroup. The remaining microvariant alleles were associated with the J2a1 and E1b1b haplogroups, with proportions of three and one, respectively. Table S4 presents the distribution of haplogroups within our dataset.

### Median joining network for Yemeni population

**Fig. 3:**
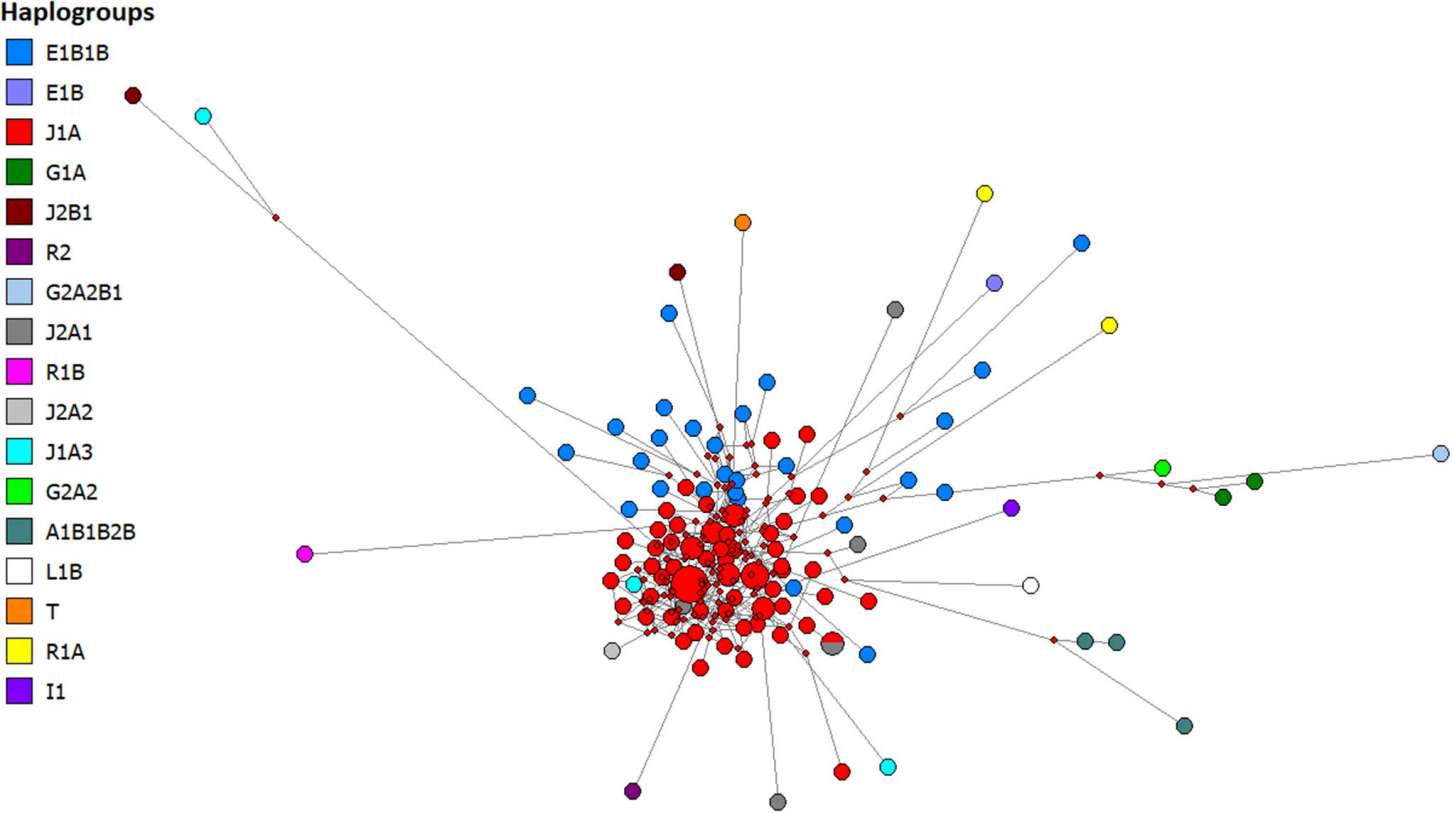
Median-joining network of Y-STR haplotypes for Yemeni population distribution of predicted haplogroups. Circles represent haplogroups, with an area proportional to sample size, and lines between them proportional to the number of mutational steps. Haplogroup categories represented in different colours are explained in the top left legend. The network’s major cluster haplogroups are assigned to J1a and E1b1b

To elucidate the interrelationships among Y-STR haplotypes in the dataset, this study constructed a median-joining network (Fig. 3) based on Y-DNA Haplogroup Predictor NevGen. Haplogroups were assigned within the network primarily based on predicted clustering. Notably, a prominent feature of the network is the presence of a central star-like cluster comprising closely linked haplogroups; wherein the red circles denote the J1a haplogroup, and the blue circles denote the E1b1b haplogroup, indicating a recent expansion of these haplogroups. The distribution of haplogroups within our dataset is detailed in Table S4.

### Populations’ structure

**Fig. 4:**
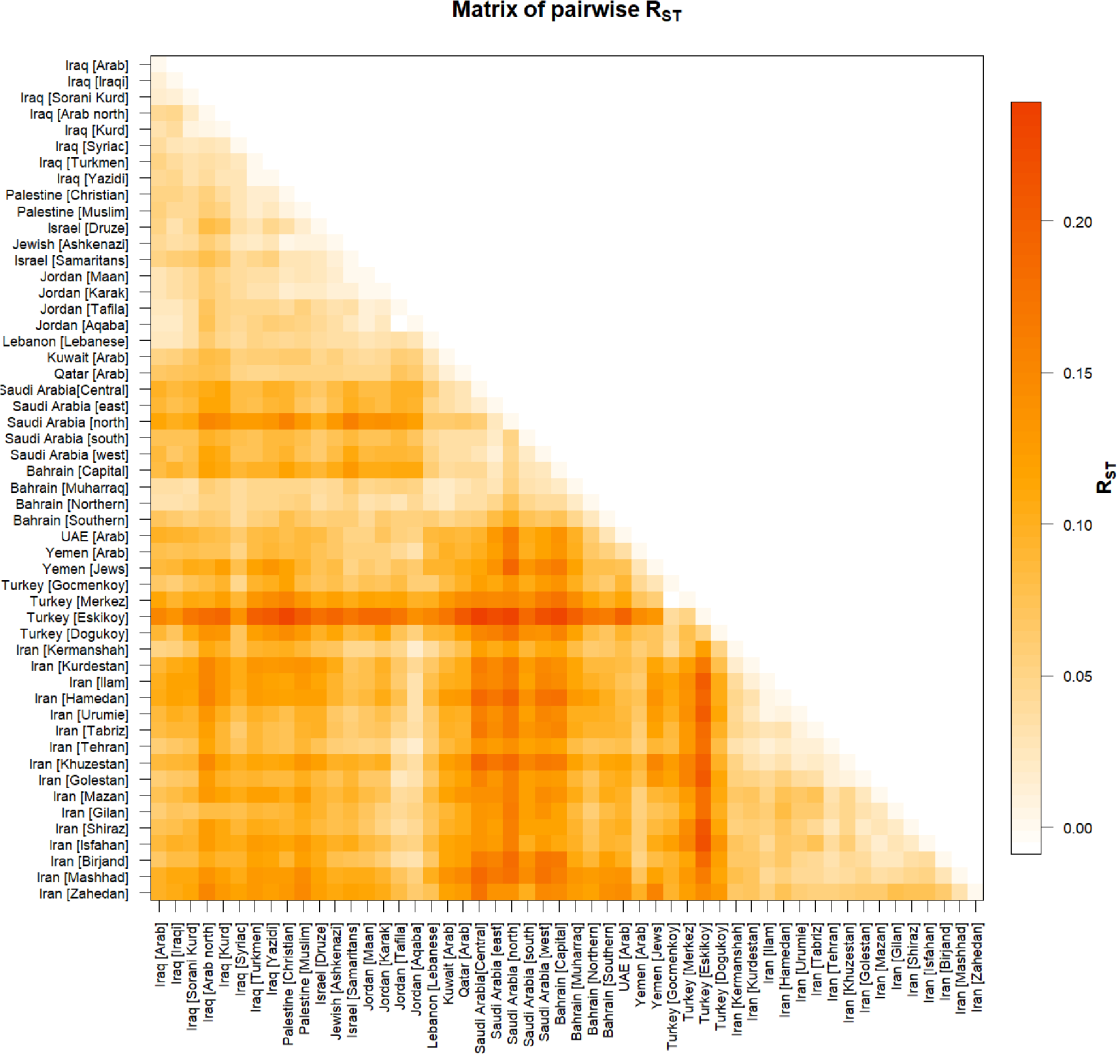
The matrix of pairwise genetic distance R _ST_ of Y-STR between the Yemeni population and the other Middle Eastern populations based on 17 Y-STR markers. The Yemeni population was compared to 52 regions and ethnic groups in the Middle East. This matrix was generated using Arlequin 3.5.2.2 software

A pairwise matrix plot of R_ST_ Distances was generated to compare the Yemeni population with 52 other Middle Eastern populations using 17 loci (Fig. 4 and S5 Table). Yemen [Arab] exhibited the highest R_ST_ value of 0.15902 in comparison to Saudi Arabia [north], whereas the lowest R_ST_ value of 0.02944 was observed between Yemen [Arab] and Yemen [Jews].

The largest R_ST_ among all population compared in our study was 0.23915, between Saudi Arabia [central] and Turkey [Eskikoy] populations; and the smallest R_ST_ (-0.00993) between Turkey [Gocmenkoy] and Turkey [Merkez] populations.

**Fig. 5:**
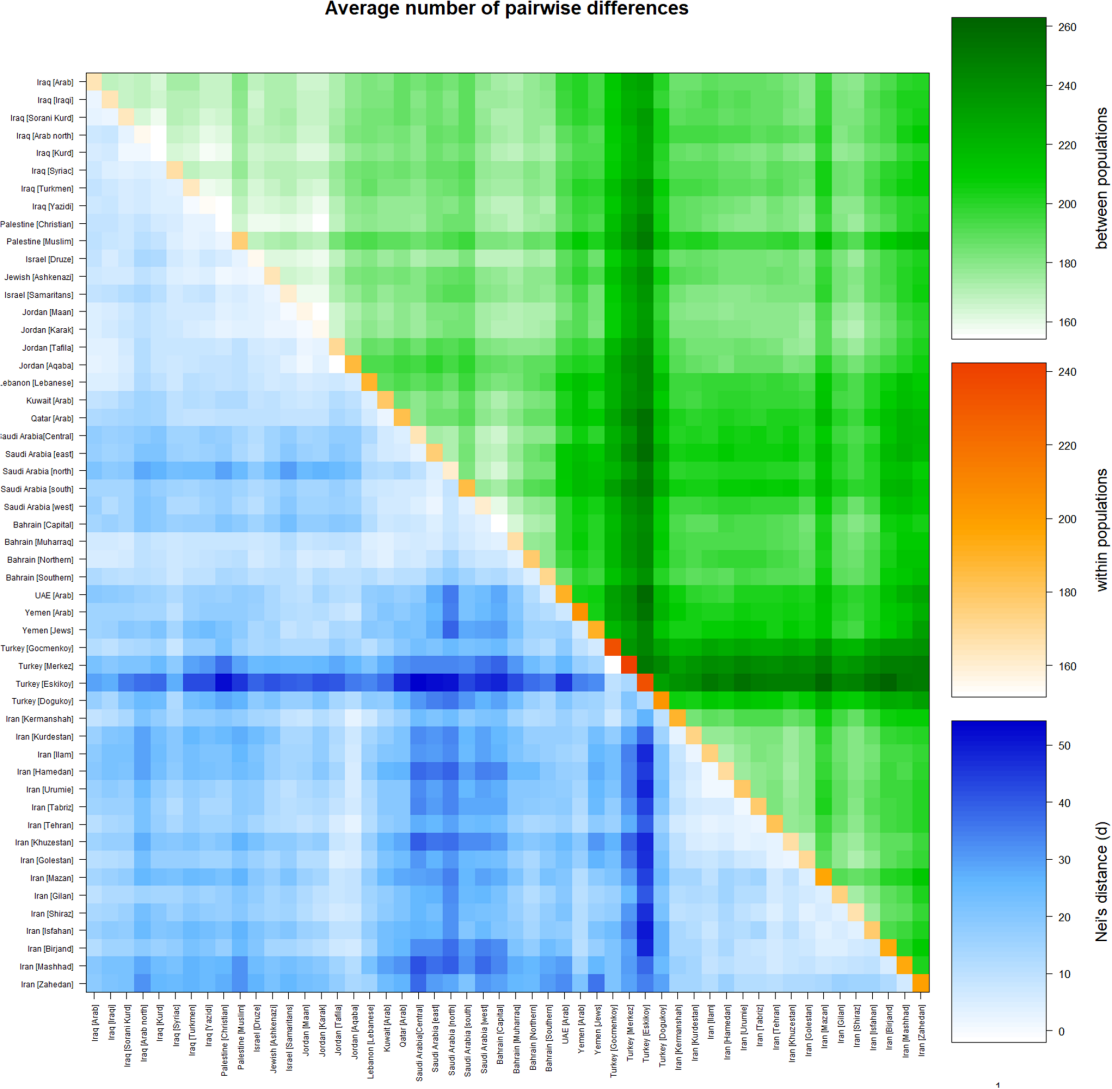
Matrix plot showing population average pairwise differences based on 17 loci in 52 Middle Eastern populations. The area above the diagonal (green) shows the average number of pairwise differences between populations (PiXY); the diagonal (orange) shows the average number of pairwise differences within population (PiX); and below the diagonal (blue) shows the corrected average pairwise difference (PiXY-(PiX + PiY)/2). The scale of differences is shown on the right side of the matrix. This matrix was generated using Arlequin 3.5.2.2 software

The average pairwise differences among 52 Middle Eastern populations were calculated using 17 loci. These average pairwise differences were calculated to show the genetic differences between and within these populations, in addition to among populations using Nei’s distance (Fig. 5 and S6 Table).

The average number of pairwise differences within the Yemeni population was calculated to be 205.13314. Among the populations under study, the highest average value of 242.23908 was observed in Turkey [Merkez], whereas the lowest average value of 151.41799 was recorded in Bahrain [Capital].

The highest average number of pairwise differences between populations, amounting to 262.93056, was observed in the comparison between the Iran [Birjand] and Turkey [Eskikoy] populations. Conversely, the smallest average number of pairwise differences (154.13004) was noted in the comparison between the Iraq [Kurd] and Iraq [Arab North] populations. Similar to the R_ST_ pattern, Yemen exhibited a parallel trend, with the highest value recorded with the Saudi Arabia [north] at 34.85265 and the lowest value observed with Yemeni Jews at 6.30257.

Consistent with the R_ST_ analysis, a similar pattern persisted, with the largest corrected average pairwise differences (54.22059) observed between the Saudi Arabia [central] and Turkey [Eskikoy] populations, and the smallest corrected average pairwise differences (-2.07274) noted between the Turkey [Gocmenkoy] and Turkey [Merkez] populations.

**Fig. 6:**
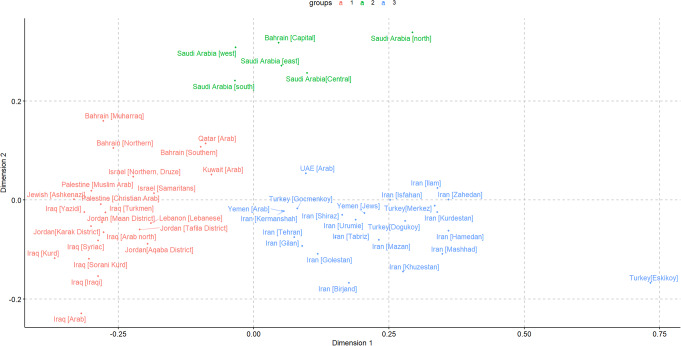
Multidimensional scaling (MDS) plots comparing the results of Yemeni and Middle Eastern populations based on 17 Y-STR markers. The Yemeni population was compared to 52 regions and ethnic groups in the Middle East. The stress value for this MDS plot was 9.523307. The MDS plot was generated using R statistical software version 4.0.3

The analysis of the populations using Multidimensional Scaling (MDS) resulted in the identification of three distinct population groups (Fig. 6). The Yemen [Arab] and Yemen [Jews] populations were found to cluster together with populations from Iran, Turkey, and the UAE, occupying the lower right quadrant of the MDS plot. In contrast, the populations of Saudi Arabia and Bahrain [Capital] formed a separate cluster in the upper region of the plot. The largest cluster was observed in the lower left part of the plot, comprising the remaining Middle Eastern populations. The stress value for this MDS plot was computed to be 9.523307, which is considered relatively high. Consequently, there was a necessity to generate a three-dimensional MDS to better interpret the population structure.

A three-dimensional multidimensional scaling (MDS) analysis was conducted, resulting in the formation of five distinct clusters. This analysis revealed a more refined distribution of the Middle Eastern population, enhancing the interpretability of the data. For an interactive exploration of the three-dimensional MDS model, please follow the link 10.25416/edgehill.28373267.v1.

### Phylogenetic analysis

**Fig. 7:**
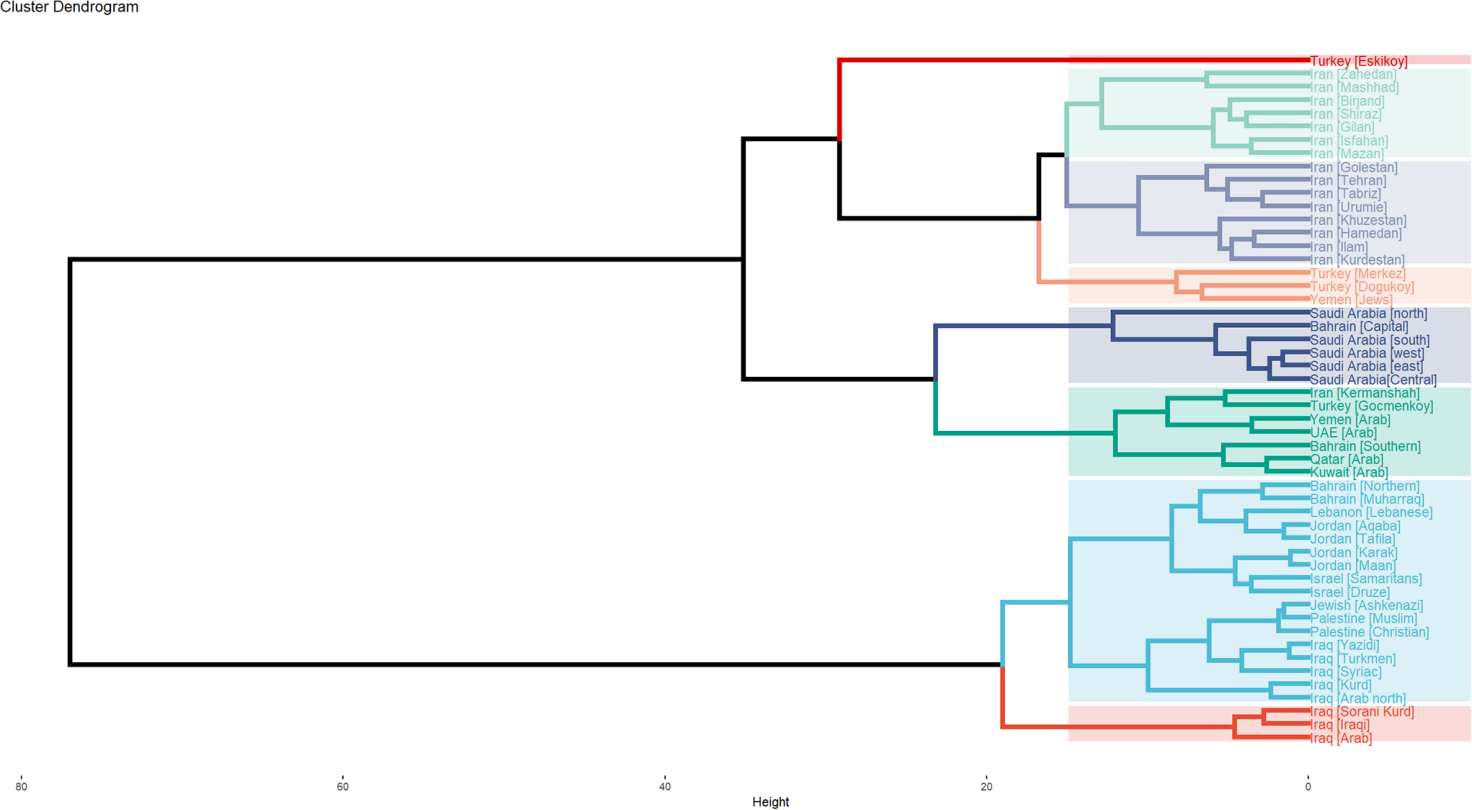
Cluster Dendrogram of the 52 Middle Eastern populations. Eight clusters (K = 8) were created. Yemen fell into one cluster with Iran [Kermanshah], Turkey [Gocmenkoy], UAE, Bahrain [Southern], Qatar and Kuwait. This dendrogram was generated using R statistical software version 4.0.3

Phylogenetic analysis of all Middle Eastern populations identified eight distinct clusters (Fig. 7). The Yemeni Arab population was grouped within a cluster that included the Arab populations of the United Arab Emirates, Qatar, and Kuwait. This cluster also encompassed the populations of Iran (Kermanshah), Turkey (Gocmenkoy), and Bahrain (Southern). Notably, the Yemeni Arab population demonstrated the closest genetic affinity to the UAE population, sharing the same terminal clade. The cluster containing the Yemeni Arab population exhibited a close phylogenetic relationship (shared a common ancestor) with various Saudi populations, which formed a separate cluster, thereby constituting a monophyletic group. Conversely, the Yemeni Jewish population was identified in a different cluster alongside two Turkish populations (Dogukoy and Merkez).

### Allelic richness

**Fig. 8:**
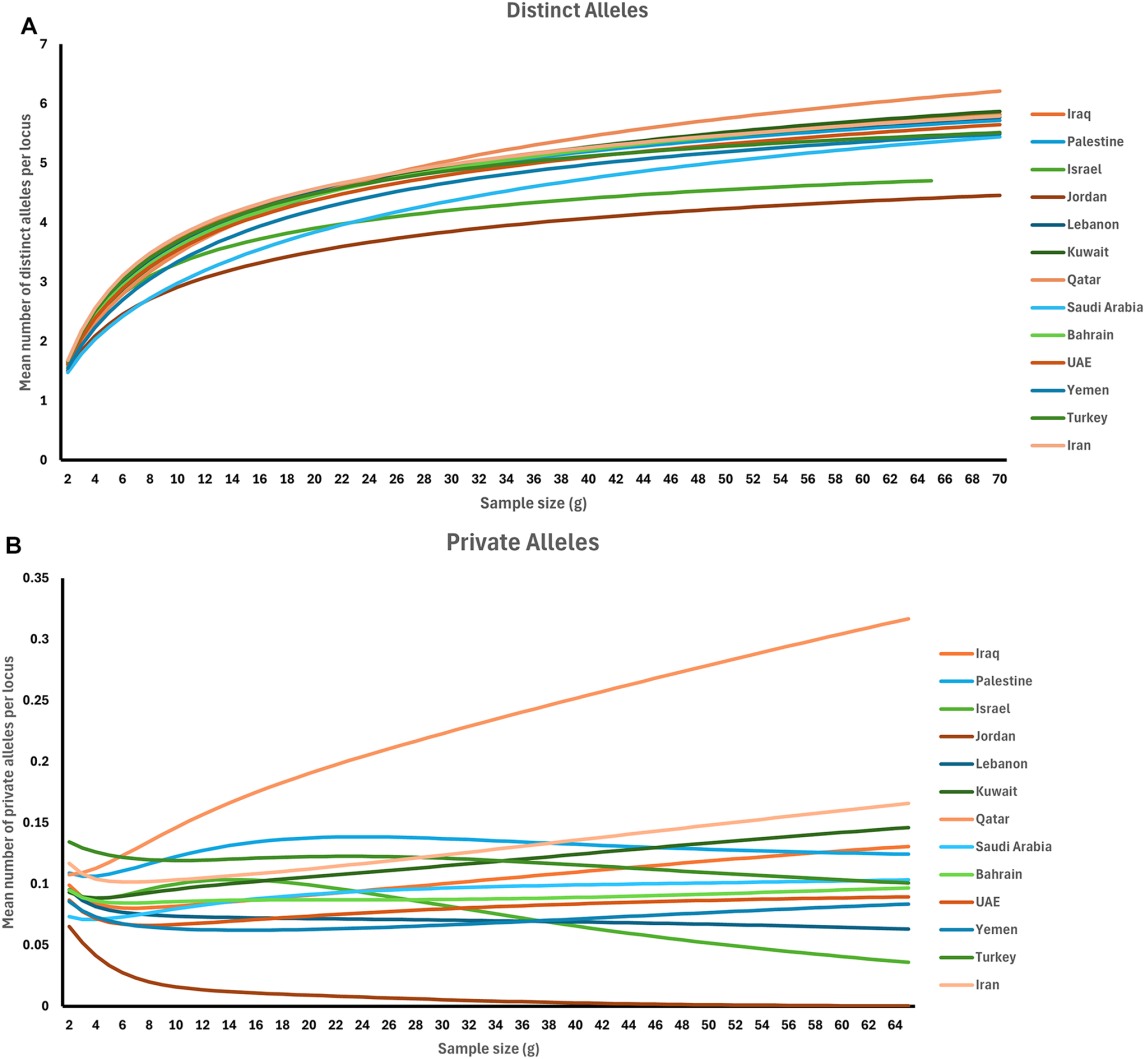
The mean number of (**A**) distinct alleles per locus and (**B**) private alleles per locus of the sixteen Middle Eastern populations

This study examined two parameters of allelic richness, specifically distinct alleles and private alleles, across Middle Eastern populations. The findings revealed that distinct alleles exhibited the highest mean values in Qatar, Kuwait, and Iraq, whereas the lowest mean values were observed in Israel and Jordan (Fig. 8A, S7 Table). Similarly, the analysis of private alleles indicated the highest mean values in Qatar, Iran, and Kuwait, while the lowest mean values were recorded in Lebanon, Israel, and Jordan (Fig. 8B, S8 Table).

### Populations’ admixture

**Fig. 9:**
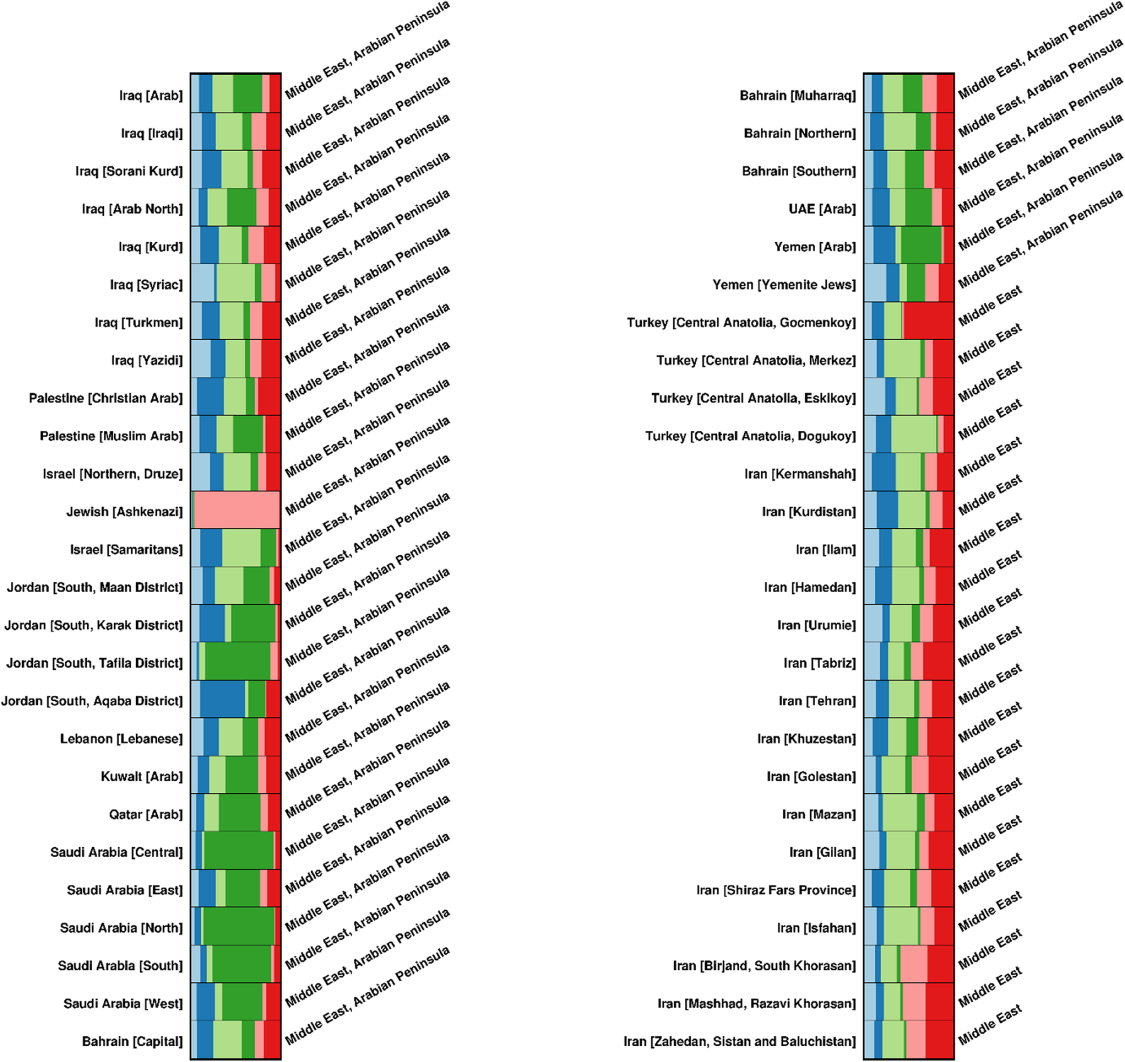
The graph of population Q- matrix of the Y-STR haplotypes using 17 STR markers from 52 Middle Eastern populations (5, 568 individuals) showing 6 clusters (K = 6)

The analysis of the Y-STR graph of the population Q-matrix, as illustrated in Fig. 9, identified six distinct clusters based on 17 STR markers sampled from 52 Middle Eastern populations, encompassing a total of 5,568 individuals. The STRUCTURE software was utilized for this analysis, employing the standard admixture model with correlated allele frequencies. S9 Table presents the population Q matrix for the 52 populations under study.

The Middle Eastern populations, excluding those from Arabia, Turkey, and Iran, exhibited genetic similarities with countries located in the northern part of the Arabian Peninsula. This includes non-Arab ethnic groups in Iraq, such as the Kurds, Syriacs, Turkmen, and Yazidis. Most of the Arabian Gulf countries, namely Saudi Arabia, Qatar, and Yemen, demonstrated a consistent genetic pattern. However, Bahrain was an outlier and clustered with the northern Arabian countries. The Ashkenazi Jewish population was the sole exception, displaying a genetic profile that significantly diverged from the other Middle Eastern populations.

### Ancestry variability

The FSTruct program was used to analyse the $\:\:{\text{F}}_{\text{S}\text{T}}/{\text{F}}_{\text{S}\text{T}}^{\text{m}\text{a}\text{x}}\:$​​ ratio of the membership coefficient variability differences between admixed and non-admixed populations, as provided by the STRUCTURE analysis. This analysis was conducted to investigate ancestry variability within Middle Eastern populations.

**Fig. 10:**
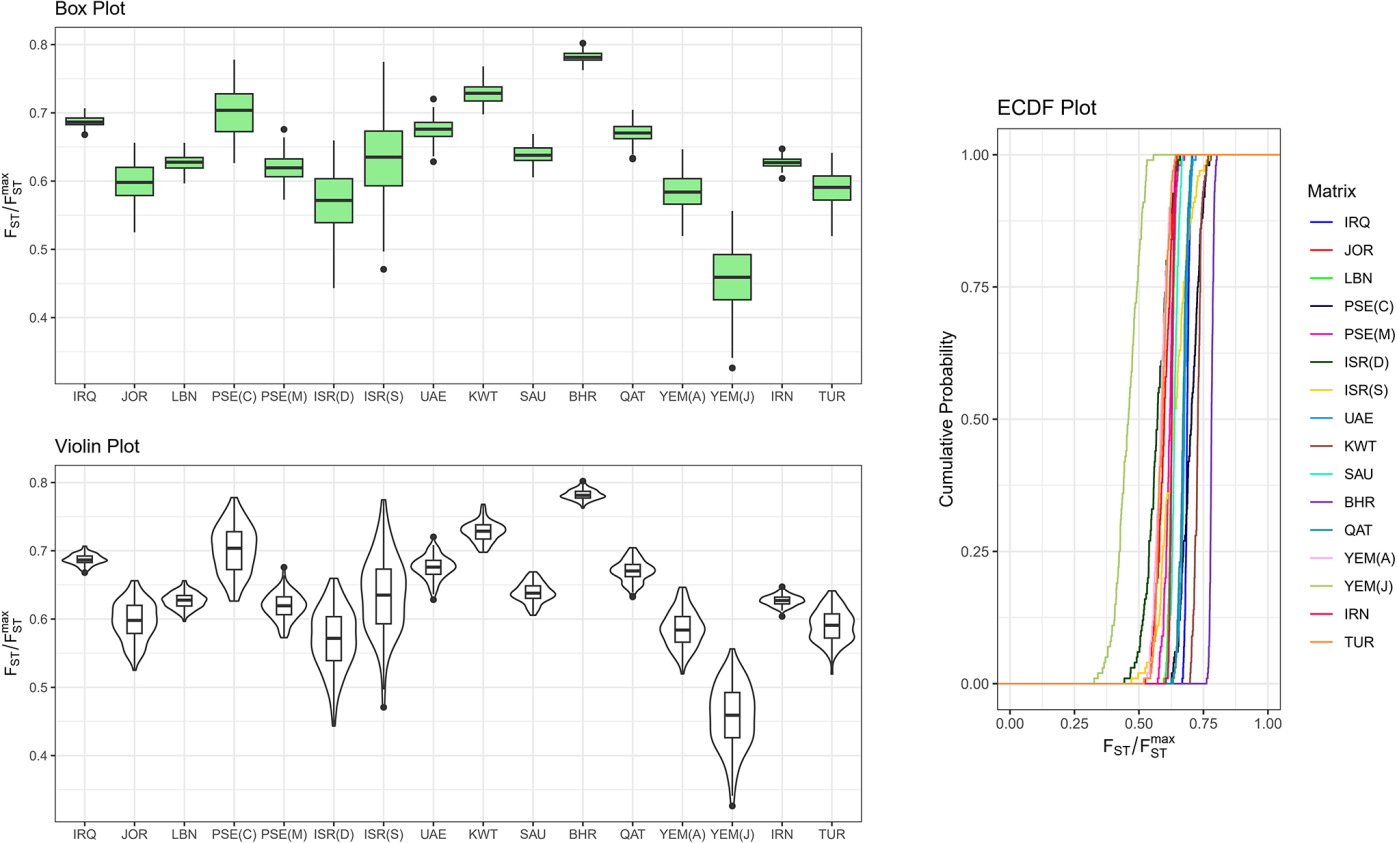
Box plot, violin plot and empirical cumulative distribution function (ECDF) plot of the bootstrap distribution of $\:\:{\mathbf{F}}_{\mathbf{S}\mathbf{T}}/{\mathbf{F}}_{\mathbf{S}\mathbf{T}}^{\mathbf{m}\mathbf{a}\mathbf{x}}$ for each Q matrix in the STRUCTURE analysis. Bahrain (BHR), Iran (IRN), Iraq (IRQ), Israel Druze (ISR (D)), Israel Samaritans (ISR (S)), Jordan (JOR), Kuwait (KWT), Lebanon (LBN), Palestine Christians (PSE)), Palestine Muslims (PSE (M)), Qatar (QAT), Saudi Arabia (SAU), Turkey (TUR), United Arab Emirate (UAE), Yemen Arab (YEM (A)), Yemen Jews (YEM (J))

The lowest $\:{\text{F}}_{\text{S}\text{T}}/{\text{F}}_{\text{S}\text{T}}^{\text{m}\text{a}\text{x}}$ ratios were observed in Yemen Jews, Israel Druze, Yemen Arab, and Turkey, with values of 0.4556665, 0.5752837, 0.5850627, and 0.5877624, respectively. Conversely, Palestine Christians, Kuwait, and Bahrain exhibited the highest ratios, with values of 0.7056174, 0.7278784, and 0.7812143, respectively. The remaining Middle Eastern regions displayed ratios ranging between 0.6 and 0.7. Detailed calculations of ancestry variability are presented in S10 Table and Fig. 10. For all statistics and ratios, as well as all statistical matrices, the Kruskal-Wallis chi-squared test indicated significant differences between the groups compared in the study (Kruskal-Wallis chi-squared = 1356, df = 15, p-value < 2.2e-16).

## Discussion

In the current investigation, Y-chromosomal short tandem repeat (STR) haplotypes of the Yemeni population were analysed using the AmpFℓSTR^®^ Yfiler™ kit, resulting in the identification of 128 haplotypes. This kit demonstrated a robust discriminative power, making it highly suitable for forensic applications.

The Yemeni population exhibits a microvariant allele frequency of 62.5% at the DYS458 marker, representing the second highest frequency identified, following Saudi Arabia at 71%. In comparison, other nations in the Arabian Peninsula exhibited lower frequencies of this microvariant allele, with Qatar at 51.1%, Kuwait at 40.1%, the UAE at 36.4%, and Iraq at 34.6%. This substantial prevalence of microvariants is particularly significant, as it is characteristic of Middle Eastern populations, with which the Yemeni population shares both a common ancestry and geographic proximity. The presence of microvariant alleles has the potential to enhance the discriminatory capacity and probative value of DNA profiling, thereby aiding in the identification of population-specific haplogroups [23].

Another notable characteristic of the presence of microvariant alleles at the DYS458 marker is their association with the J1 haplogroup, which is considered the predominant haplogroup among speakers of Semitic languages, particularly within Arab populations in the Arabian Peninsula. Numerous forensic genetics studies have documented the prevalence of haplogroup J1 in the Arabian Peninsula, including frequencies of 71% in Saudi Arabia, 48.5% in Qatar, 34.8% in the United Arab Emirates, 38% in Oman, and 36.6% in Iraq. In contrast, this haplogroup appears to be relatively scarce within various other Middle Eastern populations, such as Bahrain (23%), Lebanon (12.5%), and Turkey (8.99%) [6, 14, 22, 23, 26].

An intriguing observation regarding the haplogroups in Yemen reveals a predominance of two main haplogroups, J1 and E1b1. Specifically, J1a constitutes 59.37% of the total samples, while E1b1b accounts for 21.09%. Together, these haplogroups represent 80.46% of the total samples. This phenomenon can be attributed to a founder effect followed by genetic drift. The high frequency of non-random mating practices in the region, including a significant prevalence of consanguineous marriages, particularly among first cousins, likely contributes to this genetic pattern. These findings are consistent with previous study conducted on the genetic landscape of Yemen [6].

The Multidimensional Scaling (MDS) plots used in this study are considered the most representative and comprehensive analysis dedicated entirely to Middle Eastern populations, encompassing 52 distinct regions and populations. The largest prior study on the Middle East, conducted by Eida et al. (2023), examined 38 Middle Eastern populations [26]. Their research identified two primary clusters within the two-dimensional (2D) MDS plots. However, the inclusion of additional Middle Eastern populations in the current study resulted in the formation of three clusters, even though with a high stress value. Consequently, there was a necessity to employ a three-dimensional (3D) MDS approach to provide a more nuanced understanding of the clustering patterns among the 52 Middle Eastern populations. Furthermore, the MDS analyses employed in this study contribute to a more accurate representation of the genetic landscape in the studied regions. It helps to avoid potential biases that could arise from oversimplifying complex population structures or ignoring intra-country diversity. This level of detail is crucial for drawing meaningful conclusions about genetic relationships and population histories, particularly in regions with rich and complex demographic histories like the Arabian Peninsula. Unlike previous studies that often juxtaposed different geographical regions of their study populations with countries in the Arabian Peninsula as a whole [14, 22].

Similar to the findings from the Multidimensional Scaling (MDS) analysis, the inclusion of additional populations in the structure analysis resulted in the identification of more clusters. Specifically, the analysis in this study revealed six clusters, which is two more than the number of clusters identified in the previous study that compared 23 Middle Eastern populations [16].

Two additional investigations focused on the genetic structure of populations in the Middle East. The first study compared the Middle East with global populations and utilized 19 STR markers, identifying nine clusters despite including only seven Middle Eastern populations [23]. The second study compared the Middle East with Africa and employed 17 STR markers to analyze 38 Middle Eastern populations, through which eight clusters were discerned [26]. Both studies compared Middle Eastern populations to other ethnic groups, which has important implications for the concept of population clusters. When comparing multiple ethnicities, we would expect to observe a greater number of distinct clusters due to the increased genetic and demographic diversity represented by the additional populations. This expectation is rooted in the fundamental principles of population genetics and demographic analysis.

This study indicates that Ashkenazi Jews do not have ancestral ties to the Middle East. The data presented here support the hypothesis that the genetic makeup is predominantly of European origin. Furthermore, the findings suggest that Ashkenazi Jews have experienced a degree of isolation from other Jewish populations residing in the Middle East [45].

The observed allelic richness within the Middle Eastern population provides valuable insights, revealing significant differences among various subpopulations. However, the informativeness of this test could be substantially enhanced by incorporating a more diverse range of population samples or regions. The effectiveness of this test was demonstrated in a study on the X chromosome, where the allelic richness test was employed to assess X-chromosomal short tandem repeats (X-STR) across various and diverse regions. This test proved highly useful in evaluating the relative significance of early migrations from Africa, as well as more recent interactions with populations from the Middle East, Africa, South America, and Europe [46].

The results of the ancestry analysis conducted in this study indicate that the Yemen Arab population exhibits the lowest $\:\:{\text{F}}_{\text{S}\text{T}}/{\text{F}}_{\text{S}\text{T}}^{\text{m}\text{a}\text{x}}$value among the Arab populations in the Arabian Peninsula. This observation suggests a lesser degree of geneadmixture within the Yemeni population in comparison to other Arab populations in the region. Consequently, it is feasible to posit that populations with a history of admixture may manifest increased ancestry variability when evaluating inferred cluster memberships, as opposed to non-admixed populations. Therefore, it is plausible to consider the Yemeni population as a potential origin of the Arab populations in the region. This finding aligns with prior studies that underscore the notable significance of Yemen in Arab Y-chromosome research. The evidence indicates that Yemen has exerted a discernible influence on the genetic composition of the region, thereby playing a pivotal role in elucidating the genetic history of Arab populations [6, 47, 48].

In Summary, Yemen’s role in Arab Y-chromosome studies is significant, with evidence pointing to its influence on the genetic makeup of the region and its importance in understanding the genetic history of Arab populations.

## Limitations

Two limitations warrant consideration in this study. Principally, the geographical constraint of sample collection to Sana’a introduces potential sampling bias, as this urban cohort may not adequately represent the genetic diversity across Yemen’s heterogeneous population structure. Additionally, the analytical scope was restricted to 17 Y-STR markers, which, while informative, provided relatively limited resolution of Y-chromosomal variation compared to contemporary multiplex systems with expanded marker panels. Future investigations would benefit from implementing a more comprehensive sampling strategy encompassing diverse geographical regions within Yemen to better capture the country’s genetic landscape. Furthermore, the incorporation of an expanded Y-STR marker panel, would enhance the discriminatory power and provide more robust insights into the patrilineal genetic architecture of Yemeni populations.

## Key points

1. AmpFℓSTR^®^ Yfiler™ (17-STR) haplotypes were reported for 128 Yemeni males.
2. Two predominant haplogroups among Yemenis are J1a (59.37%) and E1b1b (21.09%).
3. Geographical subregions significantly differentiate Middle Eastern populations.
4. Ancestry variability indicates that Yemen is the origin of Arabs in the Arabian Peninsula.

## Electronic supplementary material

Below is the link to the electronic supplementary material.

Supplementary Material 1

## Abbreviations

AMOVA: Analysis of molecular variance
BC: Before Christ
DC: Discrimination capacity
DNA: Deoxyribonucleic acid
F_ST_: is the proportion of the total genetic variance contained in a subpopulation
GD: Gene diversity
HMP: Haplotype Match Probability
MDS: Multidimensional scaling
MP: Match Probability
PCR: Polymerase chain reaction
PiXY: the average pairwise differences between individuals from two different populations (X and Y) providing insights into their genetic divergence
PiX: is the average pairwise difference within population X
PiY: is the average pairwise difference within population Y
R_ST_: A pairwise population genetic distance measures that incorporate the stepwise mutation process in microsatellites
STR: Short tandem repeat
STRAF: STR analysis for forensics
UV: Ultraviolet
YHRD: Y-chromosome STR haplotype reference database
